# A Biopsychosocial and Interprofessional Approach to the Treatment of Family and Intimate Partner Violence: It Takes a Village

**DOI:** 10.3389/fpsyt.2021.738840

**Published:** 2021-11-17

**Authors:** Ellen Poleshuck, Marsha N. Wittink, Hugh Crean, Iwona Juskiewicz, Michelle A. ReQua, Catherine Cerulli

**Affiliations:** University of Rochester Medical Center, Rochester, NY, United States

**Keywords:** biopsychosocial, interprofessional teams, medical-legal partnership, stakeholder engagement, family and intimate partner violence and abuse

## Abstract

Family and intimate partner violence and abuse (FIPV) is a critical public health problem with repercussions for mental and physical health. FIPV exposure also is associated with social difficulties such as low socioeconomic status, legal issues, poor access to employment and education, housing instability, and difficulty meeting other basic needs. As a biopsychosocial problem, one discipline alone cannot adequately address FIPV. While individuals who experience FIPV traditionally seek respite, care and safety through domestic violence shelters, social services or courts, they also often present to health care settings. Building on the medical-legal partnership model with critical input from a community advisory board of individuals with lived experiences of FIPV, we implemented a person-centered approach in the health care context to cohesively integrate legal, safety, social, psychological and physical health needs and concerns. The purpose of this paper is to describe the Healing through Health, Education, Advocacy and Law (HEAL) Collaborative for individuals who have experienced psychological abuse, physical abuse, sexual abuse, or neglect related to child maltreatment, intimate partner violence, and/or elder abuse, and review our real-world challenges and successes. We describe our interprofessional team collaboration and our pragmatic biopsychosocial framework for bringing together: professional and stakeholder perspectives; psychological, medical, legal, and personal perspectives; and clinical, evidence-based, and educational perspectives. There is no doubt that creating a program with biopsychosocial components like HEAL requires professionals appreciating each other's contributions and the need to begin working from a common goal. Furthermore, such a program could not be successful without the contributions of individuals with the lived experience we seek to treat, coupled with the external health care clinicians' input. We describe lessons learned to date in an effort to ease the burden for those seeking to implement such a program. Lessons include HEAL's more recent clinical adaptions to serve patients both in-person and via telehealth in the wake of COVID-19.

## Introduction

Individuals experiencing family and intimate partner violence (FIPV), defined as child maltreatment, intimate partner violence, and elder abuse (1), frequently interact with the health care system for needs both directly and indirectly related to their abuse (2–4). More specifically, FIPV poses risk for worsening physical and mental health conditions, including posttraumatic stress disorder, compromised sleep, headaches, gastrointestinal disorders, birth outcomes, and myriad mental health issues (5–7). FIPV also is associated with increased risk for complex social needs, such as food, unstable housing, homelessness, legal difficulties, and unstable employment (8–10), all factors that affect not only individuals' mental and physical health but access to health care.

Unfortunately, health care professionals often do not address FIPV because the miss it, report limited knowledge, or experience a lack of options when patients provide a positive endorsement (11, 12). Most outpatient visits by patients experiencing FIPV are for non-injury-related concerns. Health care clinicians often do not ask about victimization (13, 14), while many patients do not disclose FIPV without specific inquiry (15). Reasons why patients may not share their FIPV experiences with their health care clinicians include believing it is irrelevant, disclosing it is embarrassing, or past negative experiences when sharing it with other health professionals (e.g., being told to leave their partner when that was not what they wanted). Trauma-related symptoms, such as avoidance, distrust for others, hopelessness, and emotion dysregulation, may impact patients' experiences receiving care, and consequently their care may be re-traumatizing. Examples include invasive physical exams that trigger memories of sexual assault or being evaluated for FIPV in the presence of the person who abused them, which may result in the perpetuation of abuse or cause the individual to cease seeking care altogether (16).

The US Preventive Services Task Force has recommended FIPV screening for women of childbearing ages. Other professional health associations also recommend screening for FIPV within pediatric practices, mental health, primary care and women's health settings (3, 17). Screening can increase recognition of FIPV within health care settings. For screening to be useful, however, health care clinicians need training, support, resources and services for responding to positive screens (18). When FIPV is disclosed, generally health care clinicians are unprepared to help due to limited training, insufficient time with patients, and few resources readily available (19–22). Complicating matters, as already described, patients with FIPV can present with multiple, complex health conditions. Shifting from one specialist to another, they are offered multiple interventions, while none address the FIPV that may continue to exacerbate their symptoms.

A comprehensive, biopsychosocial approach to FIPV, by definition, should incorporate health, safety and social needs and be based on the patient's priorities (23). While offering resources can inform patients about critical services available to help with FIPV, a piecemeal approach can become overwhelming, confusing and unsafe for individuals facing multiple and complex difficulties. As a consequence, patients often experience barriers to treatment engagement and health care clinicians often feel overwhelmed trying to coordinate care.

Community advocates with lived experience of FIPV approached an academic health center for help improving care, citing lack of sensitivity and fragmentation as significant barriers. They described wanting a collaborative and team-based approach within the health care context to help address their diverse needs related to FIPV. Limited evidence exists about how best to support individuals experiencing FIPV within health care. However, legal interventions can improve health. For example, when survivors sought safety via orders of protection (OP), some experienced less violence and health outcomes improvements (24, 25). A judge can issue an OP to prevent perpetrators of FIPV from contacting the individuals they abused. The judge determines that the violence surpasses a legal threshold, making behavior “illegal” under the law. Nationally, all states offer OPs through criminal or civil court, or both. Often individuals experiencing FIPV receive their health and legal care siloed, yet more than half seekingx OPs for FIPV at one court reported mental health symptoms needing assessment (26).

## Context

We established the Healing through Health, Education, Advocacy and Law (HEAL) Collaborative as a partnership between an academic health center, a domestic violence center, a domestic violence court, and a local legal aid society. HEAL is located in the Department of Psychiatry at our academic health center in Rochester, NY, and based on a medical-legal partnership (MLP) model. MLPs incorporate attorneys, health care professionals, and community partners to offer health, legal, and social services all in one location (27, 28). Hundreds of MLPs exist in nearly all US states (29). MLP evaluations document they can reduce stress and improve the wellbeing of patients who utilize them (30–32). Moreover, academic health centers with MLP's reduce healthcare costs for vulnerable patients (27).

**Figure 1:**
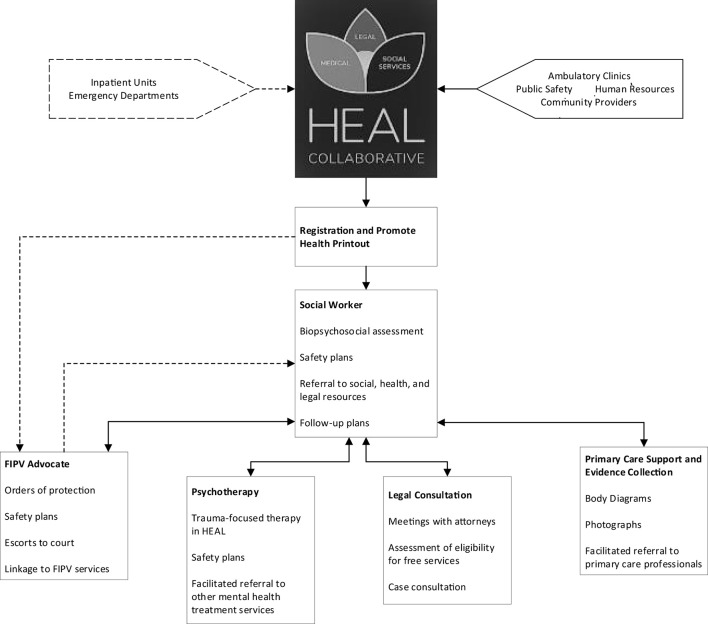
Flowchart for HEAL Collaborative. FIPV: Family and Intimate Partner Violence.

To date, MLP's primarily have consisted of legal consultants who hold clinics at primary care practices around concerns such as access to health insurance, disability applications, and eviction. We established HEAL as an MLP to offer coordinated legal assistance, social work support, trauma-focused psychotherapy, and medical consultation across the academic health center for FIPV, working in close partnership with outpatient primary and specialty care clinicians, the emergency department, and inpatient units, as well as community agencies (Figure 1). To our knowledge, HEAL is unique in three ways: (1) a focus on FIPV within the health care context; (2) the use of a discrete interprofessional team that provides support across different medical practices as well as both inpatient and outpatient settings; and (3) incorporation of stakeholder contributions as a key part of developing the program.

HEAL serves any adult in the Greater Rochester New York community coping with issues related to experiences of psychological abuse, physical abuse, sexual abuse, or neglect from child maltreatment, intimate partner violence, and/or elder abuse, with an emphasis on those served by our academic health center. We serve patients without regard to race, ethnicity, gender identity, or sexual orientation. Youth are directed to our local children's program although we work with caregivers and families. Presenting concerns range from patients who are hospitalized due to injuries caused by their partner and wanting to prevent future contact to those beginning to explore abuse in their relationships to individuals who may be safe currently but have an unresolved history of abuse. The team has a secure suite of offices within an urban hospital and is mobile within the hospital to meet individuals in crisis in the emergency department or on inpatient units. HEAL staff follow-up with ambulatory care after discharge. The HEAL consultation service ranges from urgent calls when a professional is fearful of allowing an individual to leave with a partner, caring for an individual not yet ready or able to come to HEAL, informational calls about community resources, and legal issues consultation. Our team consists of individuals with complementary expertise and skills: psychology, law, medicine, and lived FIPV experience (Table 1). This interprofessional team-based model supports a new system of care that integrates the many different needs of those who have experienced FIPV.

**Table 1:** HEAL collaborative interprofessional team members.

**Team members**		**Roles and contributions**
Direct service	Social workers	Patient assessment, planning, support and resource connection; ensuring follow-up plan is implemented; collaboration and consultation with other professionals; IPV education
	Domestic violence advocates	Patient safety planning and connection to legal protections; collaboration with other professionals; connection to emergency housing; IPV education
	Mental health therapists	Diagnostic assessment; provision of trauma- focused psychotherapy; collaboration with other professionals; facilitates psychotropic medication initiation or transferring higher level of care (partial hospitalization) or different type of care (substance use; eating disorders) when needed; assess and respond to suicide and homicide risk
	Receptionist	Screening and scheduling of patients; creates welcoming milieu by phone and in-person; administers Promote Health; tracking of program evaluation data
	Attorneys	Legal consultation and referral on IPV-related concerns (e.g., divorce, custody)
	Primary care physician	Addresses physical sequelae of IPV; evidence collection; connection with primary care
Consultative team	Psychiatrist	Consultation regarding differential diagnoses and psychotropic medication needs
	Attorney	Facilitating community partnerships; IPV legal expertise; medical-legal partnership expertise
	Clinical psychologist	Hiring, coordination and integration of team members; partnering with medical center and community; budget management; evidence-based approaches to IPV treatment
	IPV survivor advocates and Community Advisory Board	Lived experience perspectives; pragmatic application of science; IPV education

### Detail to Understand Key Programmatic Elements

The HEAL Collaborative is based on three practice foundations: (1). Creating an interprofessional team to collaborate closely to support the individual; (2). Utilizing evidencebased approaches, including using a MLP to address social needs that impact health in the context of a social determinants of health framework; and, as mentioned, (3). Employing a biopsychosocial treatment model created by our community advisory board of patient and professional stakeholders which incorporates multidisciplinary services in a health care setting supporting individuals who have experienced FIPV (23).

Our clinical team includes a social worker who provides assessment, resource connections (including occupational rehabilitation or assistance with other education and employment needs), crisis intervention, treatment planning, and support; two advocates who provide safety planning and legal advocacy for safety; two mental health therapists who offer trauma-focused psychotherapy; and a receptionist, who creates a safe and welcoming environment for patients. We also have a legal consultation clinic available for those requiring legal assistance; lawyers meet with patients to provide education and resources on issues such as housing, custody, immigration and divorce, and connect them with lawyers who can continue to work with them when appropriate for free or for a reduced fee or payments directly to the legal provider depending on the patient's financial resources. Finally, we have a primary care physician available to assess medical needs, gather evidence, and facilitate connections to trauma-informed medical care when needed. All services provided are documented in the electronic health record to facilitate communication and collaboration. In the United States, most health care, including in academic health centers, is paid for through health insurance reimbursement. While there are various types of federal (i.e. Medicaid and Medicare) and private insurance payers, billing is required for health care clinicians to be reimbursed. This billing process can generate records and notices to third parties that can jeopardize safety, for instance, if the patient's insurance is paid for by an abusive partner or family member. Moreover, adequacy of insurance coverage varies, and many patients seeking health care services face significant costs due to high deductible payments or co-payments. To address these barriers, in HEAL only the primary care physician and therapists bill for the services per health insurance regulations in the United States, while we provide our other services free of charge. Thus, we absorb the costs for the initial visits with the social workers and advocates so those initial interactions can be completed without billing, ensuring the survivor is safe and can proceed to the next step. We have assembled a broad interprofessional consultative team to support the clinical team that consists of a psychologist, lawyer, psychiatrist, and our community advisory board led by three individuals with lived experience with FIPV. The supervisory team provides training, consultation and support for challenging situations through weekly case consultation meetings, monthly supervisors meetings, quarterly quality improvement meetings, and *ad hoc* in services and acute consultations as indicated; the frequency of involvement varies by need across these roles. Soliciting the expertise of individuals with lived experience has proven a critical part of ensuring we provide practical and trauma-informed responses.

When being seen at HEAL for services as an outpatient, an individual first speaks with our receptionist who schedules the appointment and describes what to expect from the initial appointment. When they arrive, the receptionist administers Promote Health, a psychosocial screening tool administered on an electronic tablet consisting of a broad range of validated screening questionnaires to assess needs and collect descriptive data on who is using our services. This purpose of this data collection is two-fold. First, it allows us to ensure that we assess a broad range of domains known to be relevant to individuals experiencing FIPV. Second, it allows us to understand the characteristics of those using HEAL, monitor progress over time, and identify programmatic areas for improvement. Domains assessed include safety [e.g., Danger Assessment (33–35)], mental health [e.g., PHQ-9 (36); GAD-7 (37); Posttraumatic Stress Disorder Checklist (38, 39)], physical health [e.g., WHO-DAS pain items (40); sexual health], resource needs (e.g., housing, transportation, food, clothing, phone access), and barriers to treatment engagement [e.g., MEPS barriers to care (41)]. Once completed, Promote Health generates a resource list personalized to the patient's needs with services by zip code. The social worker then meets with the patient to assess goals and needs. As part of the appointment, they review the Promote Health results together. This appointment concludes with creation of a plan driven by the patient's goals and priorities; patients may or may not see the other team members depending on the plan. The social worker then collaborates with referring professionals, family members, and others involved with the patient's care, with the patient's permission.

Other HEAL team members can meet with the patient either immediately following this initial meeting or at a follow-up appointment, depending on the urgency of the needs, the patient's priorities, and the team members' availability. When a meeting with the advocate is initiated, the advocate offers safety planning, education regarding legal options, and, if needed, the opportunity to initiate an OP. When patients are unable to travel to court due to their health status (i.e. physical injuries, severe anxiety), we have state approval for the advocate and the individual to Skype with a judge to secure an OP rather than present in person. This accessibility allows substantially increased flexibility and opportunity for individuals to obtain OPs. For example, a pregnant woman admitted to the hospital for a gunshot wound by the father of her baby did not have to wait until she was discharged and could travel to court to initiate an OP. For those who can travel to court, the advocate can assist the individual in preparing the documentation needed and accompany the individual to court if desired. As a result, once the patient arrives at court, the case is expedited. The advocate provides outreach and support for the individual for the scheduled follow-up hearing two weeks later, if desired. The therapist provides diagnostic assessments and trauma-focused therapy as needed. The psychotherapists are skilled in a range of evidence-based approaches relevant for individuals who have experienced FIPV, such as Cognitive Processing Therapy for PTSD (42), Eye Movement Desensitization and Reprocessing Therapy (43), Cognitive Behavioral Therapy for Insomnia (44), and Group Interpersonal Psychotherapy for Victims of FIPV (45). For patients seeking therapy for reasons other than trauma-focused work or who may not be ready to start trauma-focused therapy, the therapists facilitate connecting them with someone else for care.

We also provide services for patients in the emergency department or inpatient units. The health care clinician identifies FIPV as a concern and reaches out to HEAL for assistance, and then the FIPV advocate travels to the patient, administers Promote Health bedside, reviews immediate legal options and resources, and initiates an OP by Skype if indicated and desired. The advocate will follow-up with the patient and unit staff for the duration of the individual's stay and assists with discharge planning. We next invite the patient to schedule a HEAL Collaborative follow-up appointment post-discharge and to consider if there are other HEAL team members who could be of assistance.

### Education

Given the interdisciplinary nature of FIPV, educational opportunities are critical to fill the gaps for professionals of all disciplines working with FIPV. We also are eager to spark commitment to addressing FIPV across interprofessional groups, especially trainees. HEAL offers introductory level didactic trainings about FIPV, as well as more in-depth research and clinical opportunities for trainees with specialized interest. Requests come from throughout our academic health center, including the emergency department; human resources; chaplaincy services; the employee assistance program; the primary care physician network; social work program; behavioral health and substance use treatment programs; physical therapy and public safety. Presentations in our community have included family court; maternal and child health outreach workers; domestic violence organizations; local public radio and television; and other community-based organizations serving people at high risk for FIPV. Professionals in multiple contexts are eager to learn how to recognize FIPV, talk about it in a constructive way, and recommend appropriate responses so they can feel competent to ask and respond. The focus of these talks is on defining FIPV, how to assess for FIPV, and providing recommendations about how to respond once it has been detected, as well as informing our colleagues about HEAL as a resource.

At HEAL, we have provided graduate and undergraduate students training opportunities across diverse fields: medicine, law, business, public health, and humanities; their experiences vary based on their educational goals and needs. A business school graduate student team created a business model to support the sustainability of HEAL. Medical and graduate students have conducted secondary analysis on data collected at HEAL and consequently authored papers and posters. Others completed medical school service hours working directly with the HEAL team to help find best practice models, compile resource options, and offer opportunities to help the team develop expansion plans. The educational need is greater than our resources, and we are seeking ways to fund a full-time educator as an additional member of the HEAL team.

### Findings

To provide an overview of who has been utilizing HEAL, we report descriptive information on a subset of 365 patients who received services and completed Promote Health surveys. These data are from a sample of convenience based on a subset of those who completed the Promote Health as part of routine care. We utilized RedCAP to collect the Promote Health data and exported the data into SPSS. We conducted descriptive statistics after accounting for missing data to identify means, frequencies, and standard deviations. The University of Rochester Human Subjects Review Board approved this project as exempt from review since it was conducted as a program evaluation.

Respondents were on average 38.6 (12.1 SD) years old. Self-reported race was: 68% Caucasian/White (*n* = 249), 24.4% African American Black (*n* = 89), 2.5% American Indian/Alaskan Native (*n* = 9), 2.2% Asian (*n* = 8), and 7.9% other (*n* = 29); 12.1% (*n* = 44) were Hispanic/Latinx. Additionally, 93.7% were cisgender women (*n* = 342), 4.9% were cisgender men (*n* = 18), and 1.4% (*n* = 5) were transgender women, transgender men, or gender-non-conforming individuals. A total of 85.8% (*n* = 313) individuals identified as heterosexual, 10.1% (*n* = 37) as bisexual, 3.0% (*n* = 11) as gay/lesbian, and 1.1% (*n* = 4) as other. Nearly half (44.9% (*n* = 164) reported being single/never married, 23.6% (*n* = 86) were currently married or living with a partner, 29.9% (*n* = 109) were divorced or separated and 1.6% (*n* = 6) were widowed. Individuals represented a range of incomes, with 56.8% (*n* = 201) reporting a total household income of < US$19,999, 21.2% (*n* = 75), US$20,000–39,999, and 22.0% (*n* = 78) ≥ US$40,000.

The mean depression score on the PHQ-9 was 9.3 (SD = 8.6) (score ≥ 10 indicates a likelihood of major depressive disorder), and 32.9% (*n* = 120) reported suicidal or death ideation; mean anxiety score on the GAD-7 was 8.4 (SD = 7.8) (score ≥ 10 indicates at least moderate anxiety); and mean PTSD severity on the PTSD Checklist was 13.2 (SD = 10.6) (score ≥14 indicates active PTSD). The mean Danger Assessment score was 7.8 (9.3 SD) (score >8 indicates significant risk of being harmed or killed).

Given the mean age of 38.6 years, we have determined that we need greater outreach to educate older people about the HEAL Collaborative resource. Such venues might include community programs targeting older adults, faith communities, local recreational clubs, and social services agencies, such as our county welfare agency, who all provide services to older populations. Our sociodemographic characteristics regarding race and ethnicity largely mirror our county composition. We are also underserving men. We see the need to do additional outreach to male serving organizations, such as fraternal agencies, local business communities, and local unions.

The substantial risk of danger highlighted the need for us to ensure our suite is located in a physically secure environment and to develop procedures to maximize the safety of our patients and our staff. Our mental health findings suggest nothing new – many of our patients show symptoms worthy of clinical assessments for posttraumatic stress disorder, anxiety and depressive symptoms. Also not surprising, but nonetheless concerning, is the high rates of suicidal or death ideation. It is common for abused individuals to feel hopeless in the face of abuse. However, our community advisory board members believe it is the ability to partner with myriad agencies though a collaborative that can restore hope. The journey was not easy – but is demonstrating success.

Initially, we identified many system barriers to implementation. Our system barriers were identified through biweekly case conferencing meetings, administrator meetings, supervisor meetings, our community advisory board meetings and patient feedback. The thoughts reflected below are the themes which resurfaced as agreed upon by the authorship team. The academic health center administration was hesitant to embrace HEAL as a program initially, particularly given hesitancy to view FIPV as a focus as well as potential financial costs and other risks involved. Safety needs required close partnership with the health center's public safety department. Yet many clinicians were already seeing these patients without knowing violence was an issue. Privacy and legal concerns by community FIPV agencies to have the HEAL clinicians record their work in the electronic health record and collaborate closely with other professionals needed to be understood and addressed. Over time, our integration and collaboration has improved tremendously, primarily by establishing partnerships, developing trust, creating shared commitment, clarifying HEAL's identity and the team members' roles, and providing education about the roles. Areas of overlap among the HEAL team members and their skillsets (e.g., safety planning; supportive counseling) created some tension early on and clear workflows and roles and responsibilities were established. In addition to writing notes to document the visit in the electronic health record, we have an internal electronic referral form in the electronic health record making it easier for clinicians in our health care center to refer to HEAL. Lastly, we have a protocol with public safety to promote the safety of our patients served and the HEAL team. Each of these steps requires ongoing negotiation, as well as evolving our shared values and commitment with interdependent roles. When the 21^st^ Century CURES Act (46) was implemented entitling all patients to have immediate access to their electronic record health documentation, we developed a process to assess if it was safe for patients to have notes released for them to view and/or ensure it was not triggering of their symptoms so we could offer the option to opt out of having their notes available when indicated.

The greatest ongoing challenges with HEAL have involved the culture shifts needed for this innovative endeavor for which no template exists; determining financial sustainability given that most of our services are not billable within existing health insurance structures; and developing our own realistic expectations. Not surprisingly, these issues are all ongoing. Given that addressing FIPV is not typically considered within the domain of health care, several years of meetings and negotiations were required with our New York State legal system, local court, institutional counsel's office, the privacy office, local service and FIPV advocacy organizations, and the University of Rochester Department of Psychiatry, housing HEAL. As laws and funding streams change, so do the parameters within which we must function. Within all this other work, the challenge of developing a sustainable business model for HEAL remains. Reimbursement for psychotherapy is not covered for treatment of a diagnosis “domestic violence.” Not all individuals meet criteria for a physical or mental health diagnosis and likely would not appreciate completing a diagnostic interview if they are not interested in psychotherapy. Further, a sizeable proportion of those we serve cannot safely use their health insurance for risk of others finding out they are seeking care. Relying on billable activities to generate revenue to cover the services for which we do not bill also limits the ability to expand our educational and clinical services. For example, we would like to be able to provide more educational and prevention activities. We are exploring ways to ensure HEAL can be self-sustaining outside of a fee-for service model. Success in this area will be essential to the continued survival of HEAL.

We have experienced other challenges at HEAL. Staff and patient safety was highlighted as a priority when we discovered one patient brought a weapon with her to her sessions and another was being stalked by a dangerous ex-partner. We continue to partner closely with public safety to develop and update our physical space, policies and procedures (e.g., locked access; checking in weapons with the public safety office; instructing patients to park in distant lot and leave their phone in the car if needed). Complex situations such as bidirectional abuse or those seeking court-mandated care challenge our understanding of who we should support. We ultimately decided we welcome anyone who self-identifies as having experienced FIPV and that it is not our team's role to judge the validity of patients' narratives.

COVID-19 also presented challenges to how we offer care at HEAL, as it did to all health care and FIPV service delivery. Our priority was to find ways to make our services available while protecting individual and staff safety and health. We initially moved all of our outpatient services to telehealth and developed a script to assess each individual's access to privacy at the initiation of each appointment. We provided education and support to other programs about how to consider safety while delivering telehealth services. We also started to offer services in the emergency department and inpatient units virtually via electronic tablet. We learned to administer Promote Health virtually in advance of scheduled appointments (although it has been completed less consistently due to implementation barriers). Over time we have shifted to a hybrid model, offering both remote and in-person visits depending on patient preference, current risk status related to the pandemic, and the safety and access of telehealth intervention. In our hybrid model, outpatients are invited (but not required) to attend their initial visit on-site so we can better assess safety and then develop an in-person or remote follow-up plan based on that particular individual's needs. Despite an initial drop in HEAL utilization in March 2020, within two months we resumed our initial rates, and referrals have continued to increase. We find many individuals have benefitted tremendously from the increased flexibility and access offered by remote appointments. Yet we continue to struggle with how to meet the growing demand and intensity of our community's needs.

As part of a larger study, we interviewed individuals who had utilized HEAL prior to COVID-19. The Short Explanatory Model Interview [SEMI (47)] is a semi-structured questionnaire developed by Lloyd and his colleagues that includes a coding manual and is based on Kleinman's theory on Explanatory Models (48). We used the SEMI to ascertain HEAL patients' explanatory models with relation to FIPV. We audiotaped and transcribed the interviews into Word documents and an interdisciplinary team analyzed the results documenting patients' HEAL experiences. Using a codebook based on the SEMI, we analyzed the transcribed interviews from HEAL patients. Preliminary findings document two extraordinary things: there were no negative patient testimonies and patients overwhelmingly remarked how exceptional their HEAL experiences were. Two illustrative examples follow:

“[It]'s a really great place for women to go, and deal with their trauma. It's a really nice setup, ***they have the legal and the social work, and the counseling, all in one*.** And it's a kind of secure place,….it's been just a really positive kind of experience. They've always been help.”

“Like they literally tried every avenue they could and you know like I said even [therapist] went above and beyond, you know I won't go through the whole story but ***she even contacted public defenders in that county and legal aid so she actually got me my first lawyer***.”

The SEMI interviews revealed that HEAL's team-based approach, accepting stance by the clinicians, and access to a physically and emotionally safe space were of particular importance to the HEAL patients interviewed.

## Discussion

We have learned individuals experiencing FIPV and their health care professionals are eager to utilize the services offered at HEAL. We receive frequent acknowledgments of the gaps HEAL is filling from individuals, health care clinicians, and community organizations. Examples of some feedback include: “This has been a very challenging case and appreciate everyone's help!”, “Thank you so much for this thorough reply - I really appreciate all of these ideas!”

We have come to appreciate the need for patience, time-intensive conversations, and, most importantly, a spirit of shared dedication to create a team that can offer services that are accessible, supportive and responsive to those who need them. Bringing this spirit of shared goals allows us to struggle together to attain meaningful consensus while capitalizing on our unique skills and strengths.

Having perspectives from individuals with lived FIPV experience was critical to the development and implementation of HEAL. For example, we debated the competing values of protecting privacy of individuals who have experienced FIPV by not sharing clinical information with other professionals vs. using a collaborative team approach to facilitate coordinated and trauma-informed support. We were able to turn to our colleagues with lived experience and ask them to describe the merits and risks of each of these approaches. Through our conversations together, these stakeholders emphasized the need for their health care professionals and others to understand their full range of needs within the context of carefully obtained informed consent. We were able to reach consensus that allows for close collaboration with the interprofessional team for individuals who do provide consent. Our stakeholders also participated in a discussion about safety and information about the level of detail and language that would be included in their electronic health record or other communication.

### Acknowledgment of Any Conceptual Or Methodological Constraints

The findings and discussion here are based on clinical data collected for treatment, safety decisions, and program evaluation. Moreover, they do not include outcomes. We are eager to take the next steps to conduct a longitudinal evaluation of HEAL's effectiveness to determine if it is replicable and scalable. Using lessons learned from HEAL, we can move toward laying the groundwork for broad dissemination and implementation.

### Conclusions

Individuals experiencing FIPV present at courts, shelters, health care centers, and social service agencies which maintain separate data collection systems and strict confidentiality mandates. In the mental health setting, we tend to focus exclusively on treating symptoms related to psychiatric diagnoses, and inadvertently can neglect the social, safety and physical health factors contributing to individuals' presentations. HEAL helps to bring these disconnected systems together.

FIPV-focused work is challenging. Many of the HEAL patients have truly catastrophic life experiences. Some present with severe physical injuries that are difficult to witness and all are invited to describe their abuse experiences in detail. Outcomes are not always what was hoped for by the individuals nor their health care clinicians. A team-based approach is important not only because of the range of skills required, but also because of the shared support needed. Working as a team allows for all members to hold both the pain and the successes together. We have learned that by creating space as a team to acknowledge and address vicarious traumatization and compassion fatigue together, we are better able to sustain the work.

Launching HEAL has taken a village of individuals with diverse training, experiences and perspectives committed to the shared goal of offering effective team-based biopsychosocial care for individuals experiencing FIPV. Expanding on the MLP model to incorporate integrated interprofessional support offers a new person-centered model of care for individuals experiencing FIPV. The educational opportunities are great and continuing to develop. While laying the foundation for this work has been necessarily challenging, it has been tremendously rewarding to our interprofessional team of clinicians, researchers, and advocates. The foundation is now in place to better respond to the complex needs associated with FIPV, and to build a system of innovation and evaluation into this experience.

## Data Availability Statement

Due to the sensitivity of this being patient data, a deidentified data set supporting the conclusions of this article will be made available with the appropriate human subjects research approvals from both institutions.

## Ethics Statement

The studies involving human participants were reviewed and approved by University of Rochester Research Subjects Review Board. The Ethics Committee waived the requirement of written informed consent for participation.

## Author Contributions

MW, EP, MR, and CC contributed to conception and design of the paper. IJ organized the database. IJ and HC performed the statistical analysis. EP wrote the first draft of the manuscript. MW, MR, and CC wrote sections of the manuscript. All authors contributed to manuscript revision, read, and approved the submitted version.

## Author Disclaimer

The content is solely the responsibility of the authors and does not necessarily represent the official views of the University of Rochester or the Patient Centered Outcomes Research Institute.

## Conflict of Interest

The authors declare that the research was conducted in the absence of any commercial or financial relationships that could be construed as a potential conflict of interest.

## Publisher's Note

All claims expressed in this article are solely those of the authors and do not necessarily represent those of their affiliated organizations, or those of the publisher, the editors and the reviewers. Any product that may be evaluated in this article, or claim that may be made by its manufacturer, is not guaranteed or endorsed by the publisher.
